# Obituary Prof. Dr. med. Johann Rastetter (28th of March 1928–23rd of June 2022)—a leading German hematologist

**DOI:** 10.1007/s00277-022-04931-7

**Published:** 2022-08-03

**Authors:** Wolfgang E. Berdel, Hermann Dietzfelbinger, Bertold Emmerich, Michael Fromm, Michael Hallek, Peter A. Maubach, Jack Nisenbaum, Albrecht Reichle, Hans D. Schick, Hubert Serve

**Affiliations:** Department of Medicine A, University Hospital, Münster, Germany


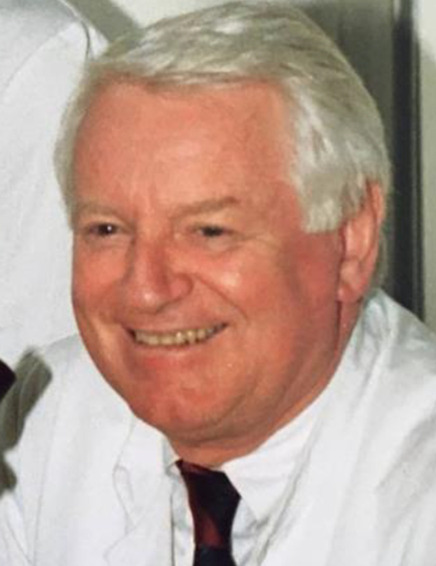


Johann Rastetter, Professor emeritus of the Faculty of Medicine, Technische Universität of Munich, Bavaria, deceased after a short and acute period of lymphoma on the 23rd of June 2022 in Munich. His final statement—still full of hope and humor—was: “What other disease could have caught me?”.

Born in Karlsruhe on the 28th of March 1928, Johann Rastetter soon moved with his family to Konstanz, where he and his older sister had a rather peaceful childhood. That changed dramatically when he was drafted at the age of 16 as a soldier in the last year of the 2nd World War. Luckily, he survived and so did his family—yet traumatized by the war forever. Since very early—already as a child—his wish was to become a medical doctor. Thus, in 1947 he started studying medicine at the University of Freiburg, a university and a city still full of destruction by the war. After finishing his exams and his MD thesis in 1952, Johann Rastetter, fascinated by blood and blood diseases, joined the Department of Internal Medicine at the University Hospital, at that time chaired by Prof. Ludwig Heilmeyer. In this department, he became board-certified for Internal Medicine in 1960 and met Prof. Herbert Begemann with whom a life-long friendship of mutual respect developed. In Freiburg, Johann Rastetter also founded his own family, with two daughters born in 1957 and 1961.

When Herbert Begemann became Director of a Department of Medicine at the Schwabing Hospital in Munich, Johann Rastetter followed as attending physician. In Schwabing besides clinical work, particularly with patients suffering from leukemia, he built a laboratory for hematological and cytological diagnostics of marrow and blood diseases. This area of hematology attracted his special attention throughout his professional life, and he also helped to develop the first generations of machinery for automated differential blood cell counts.

Together with Herbert Begemann, he assembled a group of authors, influential German hematologists at that time, and edited “Klinische Hämatologie” with the Georg Thieme Verlag. This German standard textbook for hematology was issued in sequence for several decades. Already in Freiburg, the two started to collect cytological smears and slides over the years of their cooperation, which they used to write and edit the internationally known standard color atlas with characteristic photographs and drawings of cells from blood, bone marrow, lymph nodes, pleura, ascites, and spinal fluid. The collection included cells present in the healthy organism, but in particular focused on their appearance in different entities and states of disease. Generations of young hematologists until today learn hematological cytology using this standard book entitled “Atlas der klinischen Hämatologie” published by the Springer-Verlag. It serves also as a reference for experienced hematologists in their daily practice.

In 1969, Johann Rastetter got appointed by the still young Faculty of Medicine of the Technische Universität at the Klinikum rechts der Isar in Munich, and 1970 after obtaining the qualification for a university professorship (Habilitation), he was given the task to found and build a Division for Hematology and Oncology for the Klinikum. Johann Rastetter was the director of this division until his retirement in 1997. In addition, in 1976 he was elected Vice President of the Technische Universität of Munich and filled this position for almost two decades, an exceptionally long period for an individual in such an elected office. Together with this came his responsibility as chairperson of several university boards, including the board responsible for planning and constructing modern buildings of the Technische Universität. Among the honors bestowed upon him for his work is the Federal Cross of Merit of the Federal Republic of Germany.

The authors of this obituary were associated with Johann Rastetter as students, assistant MDs, and attending physicians. Our professional development was decisively influenced by Johann Rastetter. Among his eminent characteristics was his enduring empathy with his patients, his modesty, his deep sense of humor, and his liberality towards all our ideas and ventures. To begin with the last, Johann Rastetter gave us “room to breathe” for all our initiatives in the laboratory, our translational activities and also within the field of clinical studies. When a first clinical phase I study in his division was planned in 1979 in an atmosphere for obvious historical reasons still very hesitant towards research with humans in Germany, he helped founding the first Bavarian Ethics Committee with colleagues from the Faculty, laypersons from the hospital, and lawyers from the Bavarian Ministry of Justice to provide a framework for these clinical studies according the Declaration of Helsinki. Johann Rastetter was a truly independent personality with a bit of hesitation against letting main stream dictate his thoughts and activities. At the same time, he estimated close cooperation with colleagues from other disciplines caring for cancer patients. This helped his team to found one of the first interdisciplinary Day Hospitals for the ambulatory treatment of adult cancer patients in Germany in 1984, which is still operating. He initiated interdisciplinary tumor boards very early and invited psycho-oncologist to take part in the patient care in a liaison-model. His always friendly and honest exchange with his patients helped them to keep hope. On the other hand, he did not hesitate directly facing reality in the communication with his patients, whom he regarded and respected as his partners in all decisions, an attitude of particular importance in Oncology as of course also in other fields of medicine.

1997, the year in which his granddaughter was born, was also the year of Johann Rastetter’s retirement. Finally, there was more time for his multiple interests and hobbies, such as concerts, operas, observing wild life, and traveling through Italy and Greece. Johann Rastetter devoted time painting water color pictures of his home area at the Lake Constance and his private book collection was impressive. “Hennes” as his friends called him, always was a respected source of reference for many aspects of human life.

The pandemic slowed his activities as for many of us, and finally the war in the Ukraine brought back his dark memories from the world war. His daughters told us, that he from then on refused to watch the daily news, which had been his regular habit before.

Then, in May 2022 a treatment-resistant lymphoma occurred. The fact, that his “old” nursing team in the Klinikum rechts der Isar took care of him meant great consolation for him. After a short time, while still full of plans for the future, Johann Rastetter deceased on the 23rd of June 2022 in his hospital.

Johann Rastetter is survived by his 2 daughters and his granddaughter. The field of hematology has lost an outstanding person—modest and successful. We all have lost a teacher, an eminent mentor and colleague—and an always supportive friend. Our memories and thoughts are with him—in gratitude.

